# Mechanistic Insights into *Levilactobacillus brevis* JYX2-Mediated Chinese Sauerkraut Fermentation: Nitrite Degradation and Flavor Enhancement

**DOI:** 10.3390/foods15030485

**Published:** 2026-02-01

**Authors:** Ying Ren, Weihong Tao, Wei Li, Tengda Xue, Zhijie Li, Yiwei Dai, Beiwei Zhu

**Affiliations:** SKL of Marine Food Processing & Safety Control, National Engineering Research Center of Seafood, Collaborative Innovation Center of Seafood Deep Processing, School of Food Science and Technology, Dalian Polytechnic University, Dalian 116034, China; renying010731@163.com (Y.R.); twh12345600@163.com (W.T.); lw2766420648@163.com (W.L.); lzj315121@163.com (Z.L.)

**Keywords:** *Levilactobacillus brevis*, nitrite, pilot-scale fermentation, flavor compounds, food safety, total acidity, microbial community

## Abstract

Traditional Chinese sauerkraut production is hindered by prolonged fermentation times, inconsistent quality, and nitrite accumulation during spontaneous fermentation, which collectively hinder industrial scalability. *Levilactobacillus brevis* JYX2, isolated from traditional fermented sauerkraut, exhibits excellent acid/salt tolerance, alongside distinctive metabolic pathways (for example, heterolactic fermentation, nitrite degradation, etc.), presenting a viable solution to these challenges. This study assessed its ability to improve sauerkraut quality at laboratory and pilot scales. Results indicated that JYX2 inoculation significantly reduced fermentation duration, expedited pH decline, and elevated total acid levels compared to spontaneous fermentation. The nitrite concentration in the inoculated sauerkraut was 0.72 mg/kg (*p* < 0.05), significantly lower than 1.86 mg/kg in spontaneous group. At pilot scale, nitrite levels further decreased to 0.44–0.70 mg/kg (*p* < 0.05), which is below the spontaneous group’s level of 1.61 mg/kg and approaches the “not detected” threshold (<1 mg/kg). Additionally, inoculation increased total organic acids, particularly lactic acid, and enhanced umami/sweet amino acid proportions. Microbial community analysis showed that JYX2-inoculated groups maintained higher relative abundances of core genera, including Leuconostoc and Latilactobacillus, with Firmicutes as the predominant phylum. Pilot tests confirmed consistent JYX2 performance during scale-up, with uniform quality across sauerkraut layers, showing strong industrial adaptability. In conclusion, JYX2 inoculation shortens fermentation, reduces nitrite levels, enhances flavor, and supports standardized, safe, efficient fermented vegetable production.

## 1. Introduction

Chinese sauerkraut, a representative traditional fermented vegetable product, is highly appreciated by consumers for its distinctive flavor and nutritional value [1]. Traditional sauerkraut production relies on complex microbial community succession during spontaneous fermentation, with lactic acid bacteria (LAB) serving as the dominant microbiota that generate organic acids, volatile flavor compounds, and bioactive metabolites through their metabolic activities [2]. However, this spontaneous fermentation approach exhibits inherent limitations that constrain industrial development. First, the extended fermentation cycles (typically 30–45 days) reduce production efficiency. Second, product standardization remains inadequate. Third, the process is susceptible to nitrite accumulation (‘peak periods’), contamination by spoilage microorganisms, and deterioration, raising food safety concerns. Collectively, these factors hinder the scalability and standardization of the sauerkraut industry, underscoring the need for technological innovation [3].

To address these challenges, directed fermentation technology employing functional LAB has been increasingly adopted in industrial production. Directed fermentation replaces unpredictable spontaneous fermentation with the inoculation of single or mixed starter cultures, significantly improving process controllability and product uniformity [4]. Currently, *Lactiplantibacillus plantarum*, *Lentilactobacillus buchneri* and *Lacticaseibacillus casei* have been demonstrated to improve sauerkraut flavor and shorten fermentation duration. Ren et al. (2019) reported that while *L. plantarum* contributes to flavor development, it may lead to a more standardized but potentially more uniform flavor profile [5]. Wang et al. (2024) indicated that although *L. buchneri* effectively reduces nitrite content and enhances sourness, excessive acid production can result in vegetable texture softening, decreased crispness, and growth limitations under high-salt conditions [6]. Moreover, most existing studies have been limited to laboratory-scale experiments, with insufficient validation of strain performance under industrial fermentation conditions.

*L. brevis*, as an emerging functional species within the *Lactobacillaceae* family characterized by acid and salt tolerance and unique metabolic pathways, offers novel approaches for sauerkraut flavor modulation [7]. Research demonstrates that *L. brevis* efficiently degrades nitrite and generates characteristic aromatic compounds such as phenethyl acetate at the laboratory scale while effectively shortening fermentation periods. Compared to commercial strains, indigenous strains from local fermentation systems exhibit distinct advantages in improving sensory properties, facilitating the formation of region-specific fermented products [8]. However, research on flavor differential mechanisms between this strain-dominated fermentation system and traditional sauerkraut, as well as its adaptability and stability under scale-up conditions, remains limited. Furthermore, existing achievements predominantly focus on small-scale trials, with scarce systematic correlation analyses between strain performance, process parameters, and product quality at pilot scale, severely constraining industrial application progress [9].

Therefore, this study systematically investigated the effects of *L. brevis* JYX2, isolated from traditional fermented sauerkraut and preliminarily screened in the laboratory for its nitrite-reducing capability, on fermentation processes and final product quality through combined laboratory-scale and 150-ton pilot-scale experiments. Samples from different fermentation stages were collected to determine physicochemical parameters, nitrite content, flavor compound composition, and microbial community structure. The study focused on evaluating the strain’s functional performance in shortening fermentation cycles, reducing nitrite levels, and stabilizing product quality, while validating performance consistency across production scales, thereby providing a theoretical foundation and technical support for industrial application of *L. brevis* JYX2.

## 2. Materials and Methods

### 2.1. Materials and Reagents

*L. brevis* JYX2 (highly efficient nitrite-degrading bacterial strains isolated from traditional fermented sauerkraut were identified through 16S rDNA sequence analysis as *Levilactobacillus brevis*) was preserved at the National Engineering Research Center of Seafood (Dalian, China). Fresh Yellow-Hearted Chinese Cabbage was purchased from local markets in Dalian, China. Commercial sauerkraut, designated as the Market Sales Team (MST), was procured from local supermarkets in Dalian, China.

De Man, Rogosa and Sharpe (MRS) broth and MRS agar were purchased from Qingdao Hope Bio-Technology Co., Ltd. (Qingdao, China). N-(1-naphthyl)ethylenediamine dihydrochloride, phenolphthalein, sodium nitrite, anhydrous ethanol, and sulfanilic acid were obtained from Tianjin Damao Chemical Reagent Factory (Tianjin, China); 0.1 mol/L sodium hydroxide standard solution and methanol (High Performance Liquid Chromatography (HPLC) grade) were purchased from Macklin Biochemical Co., Ltd. (Shanghai, China). Cyclohexanone and ammonium dihydrogen phosphate were obtained from Aladdin Reagent Co., Ltd. (Shanghai, China). All reagents used were of analytical grade unless otherwise specified.

### 2.2. Sauerkraut Fermentation Process

#### 2.2.1. Laboratory-Scale Strain Preparation and Inoculation

Strain activation and seed culture preparation: *L. brevis* JYX2 preserved at −80 °C was subcultured twice on MRS solid medium for activation. Single colonies were subsequently inoculated into MRS broth and incubated in a biochemical incubator (37 °C, 200 rpm, 12 h; THZ103B, Shanghai Yiheng Scientific Instruments Co., Ltd., Shanghai, China). Following incubation, the bacterial culture was harvested by centrifugation (8000 rpm, 10 min, 4 °C) using a high-speed microcentrifuge (T11A34-0248 rotor, Hitachi, Tokyo, Japan). And then, the supernatant was discarded, and the cell pellet was resuspended in sterile phosphate-buffered saline (PBS). The washing procedure was repeated three times to remove residual medium components, with final resuspension in PBS to obtain the seed culture.

Strain inoculation at different scales: In laboratory-scale sauerkraut fermentation, the prepared *L. brevis* JYX2 seed culture was inoculated to achieve a final concentration of 10^6^–10^7^ CFU/g. For pilot-scale experiments, *L. brevis* JYX2 freeze-dried powder (average viable count: 1.3 × 10^11^ CFU/g) produced by Sichuan Yidongyuan Biotechnology Co., Ltd., Meishan, China, under entrustment. Based on laboratory-scale inoculation levels (10^6^–10^7^ CFU/g), the powder was added to a 150-ton factory fermentation tank to maintain equivalent bacterial concentrations.

#### 2.2.2. Sauerkraut Fermentation

Laboratory-scale fermentation: Fresh Chinese cabbage was sun-dried outdoors for 3 days to remove excess moisture, followed by the removal of damaged leaves, while keeping intact whole heads. Two experimental groups were established: laboratory natural fermentation (LNF) and laboratory inoculated fermentation (LYF). For each group, intact whole heads of cabbage (after removal of damaged outer leaves), totaling 600 g, were placed in 2.5 L fermentation jars pre-sterilized by boiling water and cooled to room temperature, with the addition of 1200 g sterile 3% (*w*/*v*) saline solution. The LYF group received target strain inoculation at 10^7^ CFU/g with thorough mixing; the LNF group received equivalent 3% saline only. After sealing and maintaining water seals, fermentation proceeded at below 15 °C for 30 days. Samples were collected on days 0, 3, 5, 7, 11, 15, 20, 25, and 30, and then stored at ultra-low temperatures of −80 °C for subsequent analysis [10].

Pilot-scale fermentation: A 150-ton pilot-scale experiment was conducted at a sauerkraut production facility, establishing pilot natural fermentation (PNF) and pilot inoculated fermentation groups. Following proportional scale-up from laboratory procedures, approximately 50 tons of cabbage and 100 tons of sterile 3% (*w*/*v*) saline were used. The inoculated group received *L. brevis* JYX2 at 10^7^ CFU/g, with a total fermentation duration of 6 months. Due to the closed fermentation system, sampling occurred only at fermentation completion. To assess the spatial uniformity of inoculation and fermentation, the characterization method for solid-state fermentation systems described by Tian et al. (2024) was adopted with minor modifications [11]. Stratified sampling was employed, specifically involving independent sampling from the upper layer (PIU) and lower layer (PIL) of the pilot fermentation tank at sampling time point, ensuring each layer was represented by three biological replicates. This design enables detection of potential vertical gradient variations in microbial distribution and metabolite concentrations.

### 2.3. Physicochemical Parameter Determination

pH was measured using a pH meter (FE-28, Mettler-Toledo International Trade Co., Ltd., Shanghai, China). Total titratable acidity (TTA) was determined by acid-base titration according to Chinese National Standard GB/T12456-2021 [12]. The following key reagents were used for the analysis of total acidity: sodium hydroxide (Sinopharm Chemical Reagent Co., Ltd., Shanghai, China); potassium hydrogen phthalate for standardization (Guangfu Fine Chemical Research Institute, Tianjin, China); phenolphthalein indicator (Damao Chemical Reagent Factory, Tianjin, China); and anhydrous ethanol (Fuyu Fine Chemical Co., Ltd., Tianjin, China). Salinity was measured using a salinity meter (PAL-03S, Atago Scientific Instruments Co., Ltd., Guangzhou, China).

### 2.4. Nitrite Content Determination

The nitrite content was quantified by spectrophotometry according to the Chinese National Standard GB 5009.33-2016, using a microplate reader (Infinite M200, Tecan, Switzerland) [13]. The assay involves diazotization of nitrite with sulfanilic acid under weakly acidic conditions, followed by coupling with N-(1-naphthyl)ethylenediamine dihydrochloride to form a purple-red complex, with quantification by the external standard method [14] (the working solution was prepared at 5.0 µg/mL according to the cited national standard). For standard curve construction, solutions containing 0.0–12.5 μg sodium nitrite (0.00–2.50 mL) were prepared in 15 mL tubes. Each solution received 2 mL sulfanilic acid (4 g/L), was mixed and incubated (3–5 min), then received 1 mL N-(1-naphthyl)ethylenediamine dihydrochloride (2 g/L), was diluted to 10 mL with deionized water, and incubated (15 min, room temperature) before absorbance measurement at 538 nm against a reagent blank. The resulting standard curve equation was y = 0.0117x + 0.0361 (R^2^ = 0.9999). Sauerkraut fermentation liquid samples were filtered through a water-based polyethersulfone membrane (Φ50 mm × 0.22 μm, Jinlong, Tianjin, China), and 1 mL filtrate was processed identically for nitrite quantification using the established calibration curve [15].

### 2.5. Organic Acid Content Determination

Organic acid content was determined by high-performance liquid chromatography (HPLC) following a previously described protocol with modifications [16]. Briefly, organic acid analysis was performed using a Shimadzu LC-16 HPLC system equipped with a Shim-pack GIST C18-AQ column (4.6 mm × 150 mm, 5 μm; Shimadzu, Kyoto, Japan) maintained at 30 °C with detection at 210 nm. The mobile phase consisted of 0.01 mol/L ammonium dihydrogen phosphate buffer (pH 2.5, Phase A) and methanol (100%, *v/v*, Phase B), with a flow rate of 1 mL/min. The injection volume was 20 μL.

### 2.6. Free Amino Acid Content Determination

Free amino acid content was determined using an amino acid analyzer (LA8080, Hitachi High-Technologies Co., Tokyo, Japan). Accurately transfer 1.00 mL of fermented samples that have been filtered through a 0.22 μm permeable membrane. Add 3.00 mL of acetone to the filtrate, then allow it to stand for 5 min. Following the incubation period, the mixture was centrifuged at 10,000× *g*, 10 min (CF16RXII, Hitachi High-Tech Corporation, Tokyo, Japan). The resulting supernatant was collected and dried under a nitrogen flow. The residue was redissolved in 1 mL of 0.02 mol/L HCl, filtered again through a 0.22 μm membrane, and 20 μL of filtrate was injected for analysis.

### 2.7. Volatile Flavor Compound Determination

Volatile flavor compounds were analyzed by headspace solid-phase microextraction–gas chromatography–mass spectrometry (HS-SPME-GC-MS, 7890A-5975C, Agilent, Palo Alto, CA, USA) according to Li et al., with modifications [17]. Accurately weigh 2.00 g of the sauerkraut samples and place them in a 20 mL GC injection vial. Add 50 µL of a 50 mg/L cyclohexanone internal standard solution (purity ≥ 99%), place it in headspace vials, and incubate at 60 °C for 20 min. The SPME fiber was then exposed to the headspace for 20 min extraction, followed by transfer to GC-MS for volatile compound determination.

Chromatography Program: The oven temperature program was: initial 40 °C held for 3 min, increased to 100 °C at 5 °C/min, then to 175 °C at 3 °C/min, and finally to 215 °C at 10 °C/min and held for 10 min.

All flavor compounds were identified by comparing mass spectral data with the NIST14 database. Semi-quantification of volatile flavor compounds was conducted by the internal standard method through comparison of gas chromatographic peak areas.

### 2.8. Microbial Composition Determination

Microbial community genomic DNA was extracted from sauerkraut samples using the FastDNA^®^ Spin Kit for Soil (MP Biomedicals, Norcross, GA, USA) according to the manufacturer’s instructions. In brief, bacterial PCR amplification was performed using universal primers 27F (5′-AGAGTTTGATCCTGGCTCAG-3′) and 1492R (5′-AGAGTTTGATCCTGGCTCAG-3′). The resulting PCR products were pooled and sequenced on an Illumina HiSeq 2500 platform at Shanghai Majorbio Bio-pharm Technology Co., Ltd., (Shanghai, China). Community composition was statistically analyzed at different taxonomic levels.

### 2.9. Electronic Sensory Evaluation

Taste characteristics of sauerkraut fermentation liquid were evaluated using a TS-5000Z taste analysis system (Intelligent Sensor Technology, Inc., Atsugi, Japan) following a previously described protocol with modifications [18]. Prior to measurement, taste sensors and reference electrodes were filled with internal solution (3.3 mol/L KCl containing saturated AgCl), then sequentially immersed in reference solution (30 mmol/L KCl containing 0.3 mmol/L tartaric acid) and 3.3 mol/L KCl solution for 24 h activation. Following system self-checking according to instrument protocols, samples were placed in specialized beakers and detected at room temperature. Evaluated taste parameters included sourness, bitterness, astringency, saltiness, umami, and their respective aftertastes. Each sample was measured four times, with averages calculated from the last three measurements to ensure data reliability.

A PEN 3 electronic nose system equipped with 10 metal oxide semiconductor sensors was used to detect volatile components in sauerkraut, with modifications from Zhou et al. [19]. One gram of the sample was accurately weighed into a 50 mL centrifuge tube and sealed with sealing film. The detection process included sensor cleaning and sample measurement phases: clean air served as carrier gas at a 300 mL/min flow rate, with 60 s cleaning time, 1 s data acquisition interval, and 100 s total acquisition time. Complete electronic nose response signals were obtained from 10 sensors, with data collected using WinMuster software.

### 2.10. Data Processing

Origin 2021 software (OriginLab Corp., Northampton, MA, USA) was employed for result analysis and graphing; SPSS 20.0 software (International Business Machines Corp., New York, NY, USA) was used for statistical analysis of experimental data. Experimental data are presented as mean ± standard deviation (Mean ± SD), with statistical methods applied for significance analysis. *p*-values less than 0.05 were considered statistically significant.

## 3. Results and Discussion

### 3.1. Physicochemical Parameter Analysis

**Figure 1:**
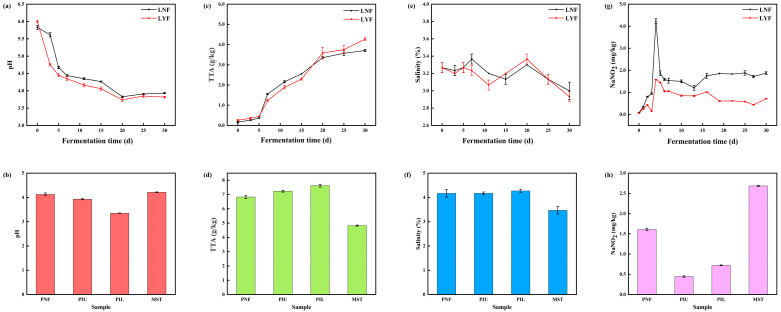
Physicochemical parameters of sauerkraut samples from different experimental groups. Changes in (**a**) pH, (**c**) TTA, (**e**) salinity, and (**g**) nitrite content during laboratory-scale fermentation. Comparison of final values of (**b**) pH, (**d**) TTA, (**f**) salinity, and (**h**) nitrite content in pilot-scale fermented sauerkraut. Abbreviations: LNF, Laboratory natural fermentation; LYF, Laboratory inoculation fermentation.

In laboratory-scale fermentation experiments, pH values in both laboratory natural fermentation (LNF) and *L. brevis* JYX2-inoculated (LYF) groups exhibited overall declining trends, with rapid pH reduction during the first 5 days. Notably, the LYF group maintained consistently lower pH values than the LNF group throughout the entire fermentation cycle (Figure 1a), indicating sustained acid-producing advantage of *L. brevis* JYX2 under laboratory-scale conditions. In pilot-scale experiments (Figure 1b), no significant pH differences were observed between the market sales team (MST) and natural fermentation (PNF) groups. However, both the upper inoculated layer (PIU, pH 3.92) and lower inoculated layer (PIL, pH 3.35) exhibited significantly lower pH values compared to PNF (pH 4.12), further demonstrating the strong acid-producing capacity of *L. brevis* JYX2. Therefore, these results suggest that inoculation with *L. brevis* JYX2 significantly accelerated sauerkraut fermentation progression, resulting in higher acidity and more complete fermentation.

In addition, the total titratable acidity (TTA)changes throughout the fermentation period further corroborate the declining pH trend [20]. As shown in Figure 1c, TTA in both LNF and LYF groups continuously increased with fermentation time during laboratory-scale fermentation, with rapid TTA elevation during the first 5 days followed by steady growth, reflecting the transition from rapid accumulation to stable generation phases. Notably, throughout the fermentation cycle, the LYF group maintained consistently higher TTA levels compared to LNF, indicating significantly enhanced acid production capacity upon *L. brevis* JYX2 inoculation. These findings corroborate the lower pH values in LYF. In pilot-scale fermentation, the inoculated lower layer (PIL) exhibited the highest TTA (7.62 g/kg), followed by the upper layer (PIU, 7.23 g/kg), natural fermentation (PNF, 6.83 g/kg), and the market sales team (MST, 4.82 g/kg). When combined with the pH results shown in Figure 1b, which demonstrate a negative correlation between TTA and pH, both PIU and PIL groups achieved more complete fermentation with superior acid production capacity compared to PNF. Collectively, these results further validate the excellent acid-producing characteristics of *L. brevis* JYX2 and its significant contribution to enhanced total acid content and fermentation optimization.

As salinity is a key determinant of microbial succession and sensory quality in fermented vegetables, salinity levels were monitored to assess potential impacts of *L. brevis* JYX2 inoculation on osmotic stability [21]. In laboratory-scale experiments, salinity changes in the LNF and LYF groups are shown in Figure 1e. Both groups exhibited fluctuating trends with slight decreases during late fermentation; by day 30, all samples stabilized within 2.9–3.0% range. Similar salinity trends between LNF and LYF indicated that *L. brevis* JYX2 inoculation did not significantly alter salinity dynamics. In pilot-scale experiments, salinity measurements of different sauerkraut fermentation liquids (Figure 1f) showed similar levels (~4%) among PNF, PIU, and PIL groups, while MST exhibited the lowest salinity (3.5%). At the 150-ton pilot scale, no significant salinity differences emerged between natural and inoculated fermentation groups post-fermentation, suggesting minimal strain impact on system salinity changes. Taken together, salinity data combined with pH and TTA results collectively demonstrate that inoculation with *L. brevis* JYX2 accelerates acidification while maintaining stable salinity in the fermentation system, thereby preserving the osmotic balance essential for product quality.

### 3.2. Nitrite Content Analysis

Nitrite accumulation is a critical food safety concern in fermented vegetables; therefore, its content was monitored throughout fermentation to assess the safety-enhancing effects of *L. brevis* JYX2 inoculation. As shown in Figure 1g, nitrite content changes during laboratory-scale sauerkraut fermentation exhibited the characteristic “nitrite peak” phenomenon during early fermentation. During this initial phase, rapid proliferation of both LAB and spoilage bacteria occurred, with spoilage bacteria-produced nitrate reductase converting vegetable nitrate to nitrite at rates exceeding enzymatic and acid-mediated degradation [22]. At the peak point, LNF reached 4.11 mg/kg while LYF contained only 0.74 mg/kg, significantly lower than LNF. Throughout the entire fermentation cycle, the LYF group consistently maintained lower nitrite than LNF. By fermentation completion (day 30), LYF exhibited significantly reduced nitrite at 0.72 mg/kg. Furthermore, in pilot-scale experiments, both PIU (0.44 mg/kg) and PIL (0.72 mg/kg) inoculated groups contained markedly lower nitrite than PNF (1.61 mg/kg) and MST (2.7 mg/kg) (Figure 1h). These results demonstrate that *L. brevis* JYX2 inoculation effectively reduces sauerkraut nitrite content at both laboratory and 150-ton pilot scales. Research indicates nitrite typically exhibits initial increases followed by decreases during sauerkraut fermentation, a pattern consistent with this study [23].

According to Chinese standards, nitrite limits (as NaNO_2_) in pickled vegetables are 20 mg/kg, with levels below 1 mg/kg considered not detected [24]. In this study, all experimental groups maintained nitrite within national standards throughout fermentation, with LYF, PIU, and PIL achieving not-detected levels. This further confirms that *L. brevis* JYX2 significantly reduces nitrite during sauerkraut fermentation. Compared to other nitrite reduction methods, the *L. brevis* JYX2 inoculation strategy employed here demonstrates unique advantages. As a natural microbial agent, *L. brevis* JYX2 efficiently reduces nitrite while maintaining traditional flavor and overall quality, providing a green and sustainable technical pathway for safe sauerkraut production.

### 3.3. Organic Acid Content Analysis

**Figure 2:**
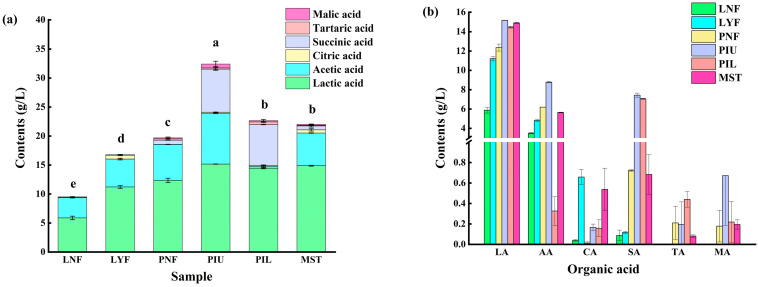
Organic acid content in sauerkraut samples from different experimental groups. (**a**) Total organic acid content and composition in sauerkraut samples from different experimental groups. Different letters indicate significant differences among groups (*p* < 0.05). (**b**) Comparison of individual organic acid contents in sauerkraut samples from different experimental groups: LA, lactic acid; AA, acetic acid; CA, citric acid; SA, succinic acid; TA, tartaric acid; MA, malic acid. Abbreviations: LNF, Laboratory natural fermentation; LYF, Laboratory inoculation fermentation; PNF, Pilot natural fermentation; PIU, Pilot inoculation fermentation upper layer; PIL, Pilot inoculation fermentation of lower layer; MST, Market sales team.

Organic acids constitute critical indicators of sauerkraut quality, directly influencing product flavor, preservation characteristics, and nutritional value [25]. A total of six organic acids were identified in sauerkraut samples, including tartaric acid, malic acid, lactic acid, citric acid, succinic acid, and acetic acid. As illustrated in Figure 2a, the total organic acid content in different experimental groups ranged from 9.51 to 32.41 g/L. Notably, all *L. brevis* JYX2-inoculated fermentation groups (LYF, PIU, PIL) exhibited significantly higher total organic acid content compared to their corresponding natural fermentation groups (LNF, PNF), indicating that strain inoculation effectively promoted organic acid synthesis and accumulation during sauerkraut fermentation.

Regarding specific organic acid composition (Figure 2b), lactic acid emerged as the predominant organic acid across all samples, accounting for approximately or exceeding 50% of the total organic acid content, followed by acetic acid. This distribution pattern is consistent with previous reports [26]. In laboratory-scale experiments, the *L. brevis* JYX2-inoculated group (LYF, 11.22 g/L) showed elevated lactic acid content compared to the laboratory natural fermentation group (LNF, 5.89 g/L). Similarly, in pilot-scale experiments, both the upper and lower inoculated layers (PIU and PIL) exhibited lactic acid contents of 15.19 g/L and 14.46 g/L, respectively, which were higher than the natural fermentation group (PNF, 12.36 g/L). These results demonstrate that this strain contributes to enhanced accumulation of lactic acid, a characteristic flavor compound in sauerkraut. Concurrently, both LYF and PIU groups displayed significantly higher acetic acid contents compared to their corresponding natural fermentation groups. Previous studies have indicated that appropriate levels of acetic acid positively contribute to enhanced storage stability and sensory quality of fermented foods [27]. Furthermore, pilot-scale fermentation groups (PNF, PIU, PIL) generally exhibited higher total organic acid contents than laboratory-scale groups (LNF, LYF), suggesting that extended fermentation duration and increased fermentation scale may provide more favorable conditions for microbial metabolism, thereby promoting further organic acid accumulation.

Lactic acid bacteria primarily produce organic acids through the metabolism of soluble sugars present in cabbage. The significant increase in total organic acid content observed in inoculated groups likely stems from the enhanced overall metabolic activity of the LAB community facilitated by the added *L. brevis* JYX2, enabling more efficient conversion of substrate sugars into various organic acids. Specifically, the elevated lactic acid content may result from homofermentative lactic acid fermentation pathways promoted by *L. brevis* JYX2 itself or by other LAB stimulated by its presence, directly driving lactic acid synthesis. Meanwhile, the increase in acetic acid may partially originate from enhanced production of acetic acid as a by-product in heterofermentative lactic acid fermentation pathways, and may also be associated with indirectly activated metabolic activities of acetic acid bacteria in the fermentation microenvironment following inoculation [28].

### 3.4. Free Amino Acid Content Analysis

**Figure 3:**
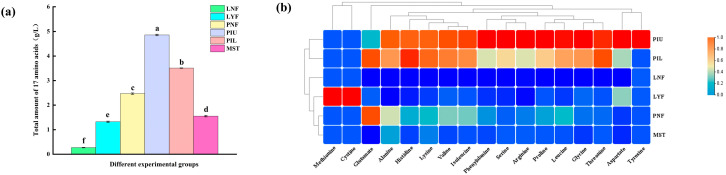
Free amino acid content in sauerkraut samples from different experimental groups. (**a**) Total free amino acid content and composition in sauerkraut samples from different experimental groups. Different letters indicate significant differences among groups (*p* < 0.05). (**b**) Heatmap cluster analysis of free amino acids in sauerkraut samples from different experimental groups.

Free amino acids serve as critical flavor precursors that interact with various taste receptors to elicit diverse taste characteristics. Consequently, the majority of free amino acids significantly contribute to the overall flavor profile of fermented foods [29]. As shown in Figure 3a, the total content of 17 detected free amino acids in the *L. brevis* JYX2-inoculated group (LYF, 1.33 g/L) was substantially higher than that in the laboratory-scale natural fermentation group (LNF, 0.27 g/L). Consistently, in pilot-scale experiments, both the upper inoculated layer (PIU, 4.85 g/L) and lower inoculated layer (PIL, 3.52 g/L) exhibited significantly higher total free amino acid contents compared to the natural fermentation group (PNF, 2.46 g/L) and market sales team (MST, 1.56 g/L). These results demonstrate that inoculation with *L. brevis* JYX2 effectively promotes free amino acid generation during sauerkraut fermentation. Regarding the taste characteristics of individual amino acids, these compounds can be broadly classified into three categories: umami-imparting amino acids (e.g., aspartic acid, glutamic acid, phenylalanine), sweet-tasting amino acids (e.g., threonine, serine, glycine), and bitter-tasting amino acids (e.g., isoleucine, leucine) [29]. As illustrated in Figure 3b, the *L. brevis* JYX2-inoculated group (LYF) exhibited significantly elevated contents of aspartic acid, phenylalanine, threonine, serine, and glycine compared to the natural fermentation group (LNF). Similarly, both pilot-scale inoculated groups (PIU and PIL) displayed the same trend, with substantially higher levels of these umami- and sweet-tasting amino acids compared to the natural fermentation group (PNF) and market sales team (MST). Notably, no significant differences in bitter-tasting amino acid contents were observed between *L. brevis* JYX2-inoculated groups and natural fermentation groups. From a sensory quality perspective, these amino acid profiles reveal important implications for flavor enhancement. The results indicate that sauerkraut inoculated with *L. brevis* JYX2 contains significantly higher total contents of umami- and sweet-tasting amino acids, which may favorably contribute to improved sensory quality. The preferential accumulation of these desirable amino acids, coupled with the absence of increased bitter amino acids, suggests that *L. brevis* JYX2 inoculation improves sauerkraut flavor by enhancing umami and sweet taste profiles, thereby producing a more palatable and delicious product compared to traditional naturally fermented sauerkraut.

### 3.5. Volatile Flavor Compound Analysis

**Figure 4:**
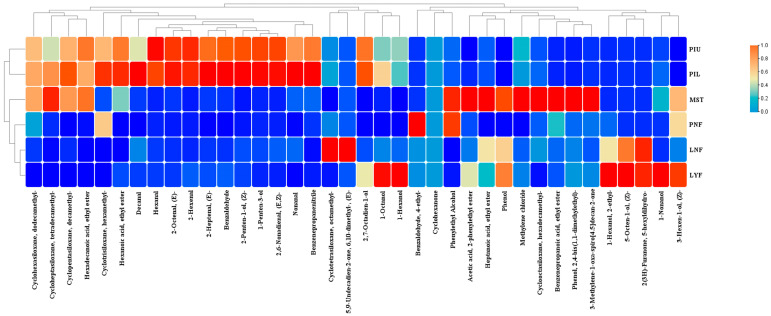
Heatmap cluster analysis of volatile compounds in sauerkraut samples from different experimental groups.

The distinctive aroma profile of sauerkraut arises from complex interactions among multiple volatile compounds, wherein isothiocyanates, nitriles, and sulfur-containing compounds are typically recognized as key characteristic flavor components. Concurrently, aldehydes, esters, alcohols, and heterocyclic compounds also contribute significantly to the overall aroma profile [30]. Through headspace solid-phase microextraction coupled with gas chromatography–mass spectrometry (HS-SPME-GC-MS), a total of 38 volatile compounds were identified across all sauerkraut samples (Figure 4), primarily comprising 8 aldehydes, 6 alkanes, 5 esters, 5 alcohols, 3 ketones, 2 phenols, minor quantities of acids, alkenes, and other compounds.

At the laboratory scale, volatile compound profiles showed similar patterns between fermentation groups. No significant differences in volatile compound composition were observed between the natural fermentation group (LNF) and the *L. brevis* JYX2-inoculated group (LYF). However, under pilot-scale conditions, distinct volatile profiles emerged in inoculated fermentations. The contents of several compounds, including cyclopentasiloxane, (*E*)-2-octenal, and cyclohexasiloxane, were significantly elevated in both the upper and lower inoculated layers (PIU, PIL). The substantial increase in (*E*)-2-octenal content imparts characteristic grassy and nutty aromas to sauerkraut, enhancing the fresh green-note aroma attributes of inoculated sauerkraut, which likely originates from fatty acid oxidation pathways [31]. Cyclopentasiloxane and cyclotetrasiloxane were ubiquitously present across all groups, though these compounds contribute minimal flavor impact. Nonanal exhibited higher contents in the pilot-scale natural fermentation group (PNF) and both inoculated layers (PIU, PIL), conferring citrus and fatty aromas to samples, likely associated with microbial metabolism-mediated lipid degradation [32].

Regarding specific aroma-active compounds, several distinct patterns emerged across fermentation conditions. The *L. brevis* JYX2-inoculated laboratory-scale group (LYF) contained 2(3*H*)-furanone derivatives and spirocyclic ketones, which typically exhibit caramel-like or coumarin-like aromas, further enriching the flavor complexity of samples. Alcohols and esters play pivotal roles in shaping the fruity and floral aroma characteristics of sauerkraut [33,34]. 1-Nonanol, 1-octanol, and phenylethyl alcohol exhibited relatively higher contents in LYF, contributing floral and sweet notes. 2-Phenylethyl acetate was also detected in the market sales team (MST), imparting honey and rose aromas to samples. Ethyl palmitate and ethyl hexanoate were detected across multiple groups, presenting creamy and fruity characteristics, respectively [35].

From an overall aroma profile perspective, these volatile compound data reveal important practical implications. The volatile flavor profiles of *L. brevis* JYX2-inoculated sauerkraut exhibited high similarity to naturally fermented sauerkraut at both laboratory and pilot scales, indicating that the inoculated fermentation process essentially preserves the core flavor characteristics of traditional sauerkraut. This preservation of traditional aroma profiles, combined with the enhanced non-volatile flavor components (organic acids and amino acids) demonstrated earlier, confirms that *L. brevis* JYX2 inoculation optimizes fermentation performance while maintaining sensory authenticity.

### 3.6. Microbial Community Structure Analysis

**Figure 5:**
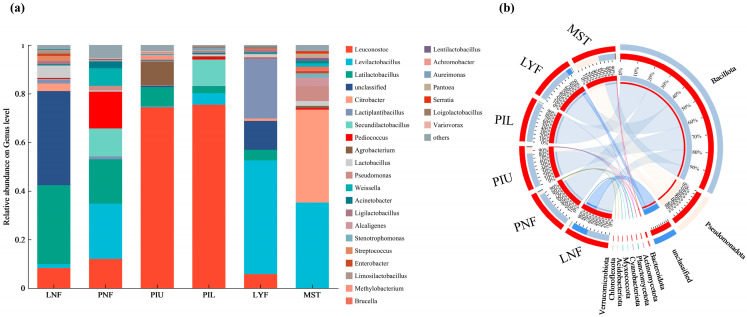
Microbial diversity analysis of sauerkraut samples from different experimental groups. (**a**) Stacked bar chart showing the composition and relative abundance of the top 30 most abundant species across all samples. (**b**) Circos diagram illustrating the relationships between samples and microbial species. The left side displays samples grouped by experimental conditions, while the right side shows dominant microbial species.

Microbial community bar charts intuitively illustrate the species composition and relative abundance in each sauerkraut sample [36]. Upon fermentation completion, all samples were predominantly colonized by *Leuconostoc* and *Levilactobacillus*-related genera (Figure 5a), consistent with previous reports [37]. In laboratory-scale fermentation systems, the natural fermentation group (LNF) was dominated by *Latilactobacillus* and *Leuconostoc* as the absolute predominant bacterial genera. In contrast, the *L. brevis* JYX2-inoculated group (LYF) exhibited distinct community structure alterations. While *Leuconostoc* maintained its dominance, the relative abundances of *Levilactobacillus* and *Lactiplantibacillus* were significantly elevated, and *Latilactobacillus* consistently maintained high abundance. These findings demonstrate that strain inoculation preserved core functional bacteria while optimizing overall community structure. Consistent with laboratory-scale observations, pilot-scale experiments revealed similar community optimization patterns. In pilot-scale fermentation systems, the *L. brevis* JYX2-inoculated groups (PIU, PIL) exhibited significantly higher relative abundances of *Leuconostoc* compared to the natural fermentation group (PNF). Furthermore, *Latilactobacillus* maintained substantial proportions in both PIU and PIL, indicating that the inoculation strategy effectively sustains the dominance of this core functional bacterium at pilot scale, thereby ensuring fermentation process stability and reproducibility.

Beyond genus-level analysis, Circos diagrams (Figure 5b) further revealed both similarities in dominant bacterial composition and significant differences in relative abundances across samples [38]. *Firmicutes* constituted the absolutely dominant phylum across all groups; however, its abundance displayed pronounced gradients under different fermentation conditions. In laboratory-scale systems, the natural fermentation group (LNF) exhibited 55% *Firmicutes* abundance, whereas the *L. brevis* JYX2-inoculated group (LYF) showed a substantial increase to 86%. In pilot-scale systems, the natural fermentation group (PNF) exhibited 90% *Firmicutes* abundance, the upper inoculated layer (PIU) showed 83%, and the lower inoculated layer (PIL) reached 97%. The market sales team (MST) displayed the lowest *Firmicutes* abundance at only 40%. From a functional microbiology perspective, these phylum-level patterns provide mechanistic insights into fermentation optimization. *Firmicutes* represents a critical functional phylum throughout the sauerkraut fermentation process [39]. In this study, *Firmicutes* abundances in all inoculated fermentation groups (LYF, PIU, and PIL) were generally higher than their corresponding natural fermentation counterparts, with the core *Latilactobacillus* genus consistently maintaining elevated abundance in inoculated groups. These results demonstrate that experimental strain inoculation optimizes microbial community structure by enriching *Firmicutes* and stabilizing the dominance of core *Latilactobacillus*, thereby more efficiently driving the sauerkraut fermentation process. We infer that this establishes a robust foundation for potential enhancements in final product quality and stability, which merits direct validation through dedicated consumer and shelf-life studies in future work.

### 3.7. Electronic Sensory Analysis

**Figure 6:**
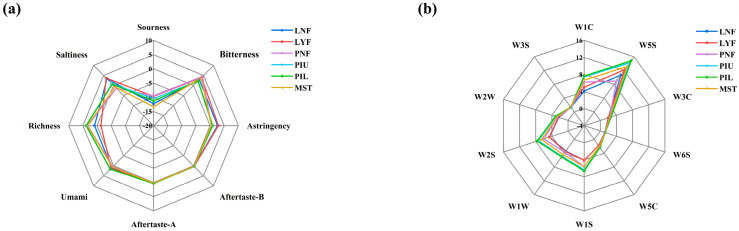
Sensory profile analysis of sauerkraut samples from different experimental groups using electronic tongue (**a**) and electronic nose (**b**).

The results of the electronic tongue radar charts of different sauerkraut samples are presented in Figure 6a. All groups exhibited minimal differences in bitterness, astringency, aftertaste-B (bitter aftertaste), and aftertaste-A (umami aftertaste) dimensions. Regarding the saltiness dimension, no significant differences were observed among groups, consistent with the stable salinity profiles demonstrated earlier. Notably, the laboratory-scale *L. brevis* JYX2-inoculated group (LYF) exhibited the highest sourness sensor response value, directly corresponding to the elevated organic acid content documented previously. The market sales team (MST) and pilot-scale lower inoculated layer (PIL) displayed superior umami and richness response values compared to other groups. In terms of overall taste profile, the pilot-scale natural fermentation group (PNF) and both *L. brevis* JYX2-inoculated layers (PIU, PIL), along with the market sales team (MST), exhibited relatively balanced profiles, primarily differentiated by sourness responses. The findings indicate that this strain primarily influences sourness and umami characteristics during sauerkraut fermentation, without significantly affecting other taste attributes. *L*. *brevis* and other LAB produce organic acids such as lactic acid and acetic acid during fermentation. Appropriate levels of acetic acid contribute distinct sour-aromatic notes to the product, with its sharp acidity complementing the mild acidity of lactic acid, collectively shaping the characteristic sour taste profile of sauerkraut [40]. Regarding umami enhancement, glutamic acid and other umami amino acids represent critical sources of savory taste in fermented vegetables. Certain LAB can degrade macromolecular substances in fermentation substrates into small molecules such as amino acids, thereby enhancing product palatability [41]. This mechanism corresponds directly to the significantly elevated umami perception observed in *L. brevis* JYX2-inoculated sauerkraut in this study, confirming the biochemical basis established in the free amino acid analysis.

Complementing the taste profile evaluation, electronic nose analysis was conducted to assess volatile compound patterns and validate the aroma characteristics. Figure 6b presents the electronic nose flavor detection response values for different experimental sauerkraut groups. All groups exhibited extremely high responses on the W5S sensor, indicating that nitrogen oxides constitute the predominant volatile compound category across all samples. Conversely, universally low responses on the W2S sensor (mostly ≤ 4) suggest minimal contents of alcohols and aldehydes in all samples. Consistent with laboratory-scale patterns, pilot-scale inoculated groups displayed distinct volatile profiles. Compared to naturally fermented sauerkraut (PNF) in pilot-scale experiments, the *L. brevis* JYX2-inoculated groups (PIU, PIL) exhibited higher response values for aromatic hydrocarbons (W1C sensor), sulfur compounds (W1W, W2S sensors), and nitrogen oxides (W5S sensor). This trend aligns consistently with laboratory-scale observations comparing the natural fermentation group (LNF) and *L. brevis* JYX2-inoculated group (LYF), further demonstrating that *L. brevis* JYX2 promotes the generation of aromatic hydrocarbons, sulfur compounds, and nitrogen oxides during sauerkraut fermentation. These volatile compounds contribute distinctive aromas to sauerkraut and synergistically interact with other flavor substances to enrich the overall flavor profile and enhance product quality.

## 4. Conclusions

**Figure 7:**
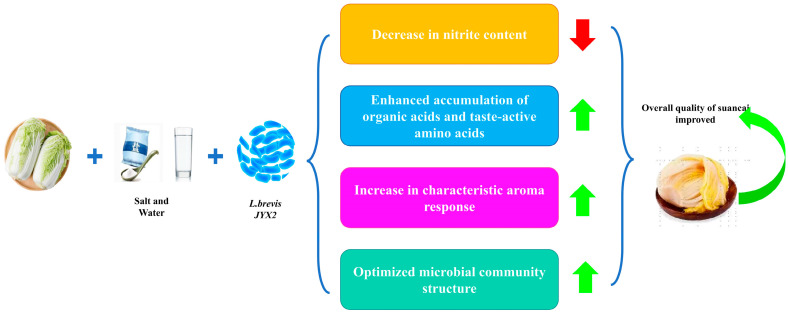
Proposed mechanism for quality improvement of sauerkraut inoculated with *Levilactobacillus brevis* JYX2.

This study provides a systematic, scale-independent evaluation of *L. brevis* JYX2, focusing on its impact on the physicochemical profile, microbiota structure, and sensory attributes of sauerkraut. Strain JYX2 accelerated acidification, rapidly reduced pH, and maintained high total acidity without perturbing salinity, demonstrating compatibility with low-salt protocols. It consistently reduced residual nitrite below the 1 mg kg^−1^ detection limit, providing a clear safety advantage. Microbial community analysis showed that JYX2 enriched *Firmicutes* and *Leuconostoc* while enhancing the metabolic potential of the core microbiota. Metabolomics revealed increased lactic and acetic acids, along with umami- and sweet-associated amino acids, enhancing the product’s “fresh-sweet” character. Although the core volatile profile remained intact, inoculated batches displayed intensified umami and key aroma notes, achieving a premium profile without sacrificing traditional identity (Figure 7). Collectively, the data demonstrate robust, scale-independent performance that simultaneously enhances safety, flavour, and process predictability, positioning JYX2 as an industrial starter candidate. Future work should aim to elucidate the metabolic pathways and interspecies interactions of JYX2 within complex fermentation ecosystems, while simultaneously monitoring biogenic amine formation. This integrated approach will provide a comprehensive safety assessment and support precise process optimization.

## Data Availability

The original contributions presented in the study are included in the article. Further inquiries can be directed to the corresponding authors.

## Abbreviations

The following abbreviations are used in this manuscript:

LNF	Laboratory natural fermentation
LYF	Laboratory inoculation fermentation (JYX2)
PNF	Pilot natural fermentation
PIU	Pilot inoculation fermentation upper layer
PIL	Pilot inoculation fermentation of lower layer
MST	Market sales team
